# Charting Nanocluster Structures via Convolutional Neural Networks

**DOI:** 10.1021/acsnano.3c05653

**Published:** 2023-10-19

**Authors:** Emanuele Telari, Antonio Tinti, Manoj Settem, Luca Maragliano, Riccardo Ferrando, Alberto Giacomello

**Affiliations:** † Dipartimento di Ingegneria Meccanica e Aerospaziale, Sapienza Università di Roma, Rome 00184, Italy; ‡ Dipartimento Scienze della Vita e dell’Ambiente, Università Politecnica delle Marche, Ancona 60131, Italy; § Center for Synaptic Neuroscience and Technology, Istituto Italiano di Tecnologia, Genova 16132, Italy; ∥ Dipartimento di Fisica, Università di Genova, Genova 16146, Italy

**Keywords:** machine learning, metal nanoclusters, collective variables, molecular dynamics, structure classification

## Abstract

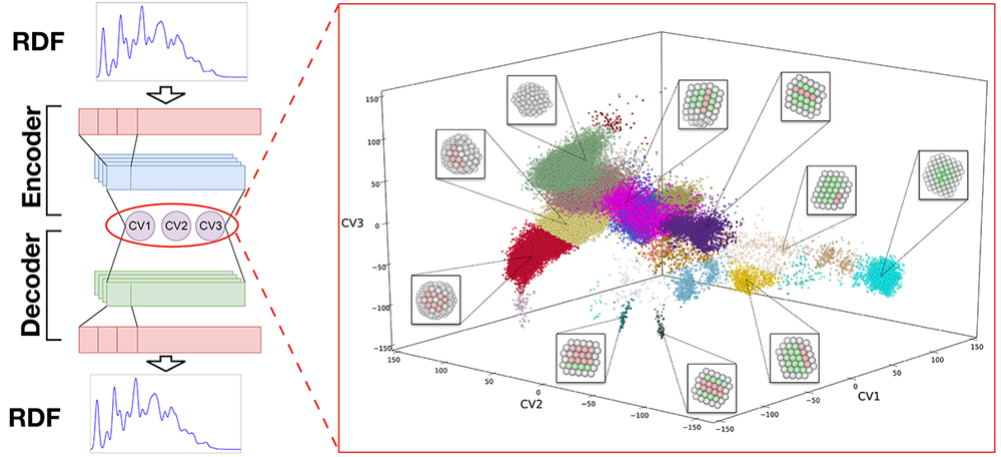

A general method to obtain a representation of the structural landscape of nanoparticles in terms of a limited number of variables is proposed. The method is applied to a large data set of parallel tempering molecular dynamics simulations of gold clusters of 90 and 147 atoms, silver clusters of 147 atoms, and copper clusters of 147 atoms, covering a plethora of structures and temperatures. The method leverages convolutional neural networks to learn the radial distribution functions of the nanoclusters and distills a low-dimensional chart of the structural landscape. This strategy is found to give rise to a physically meaningful and differentiable mapping of the atom positions to a low-dimensional manifold in which the main structural motifs are clearly discriminated and meaningfully ordered. Furthermore, unsupervised clustering on the low-dimensional data proved effective at further splitting the motifs into structural subfamilies characterized by very fine and physically relevant differences such as the presence of specific punctual or planar defects or of atoms with particular coordination features. Owing to these peculiarities, the chart also enabled tracking of the complex structural evolution in a reactive trajectory. In addition to visualization and analysis of complex structural landscapes, the presented approach offers a general, low-dimensional set of differentiable variables that has the potential to be used for exploration and enhanced sampling purposes.

## Introduction

Finite-size aggregates of atoms, molecules, or colloidal particles, can present a much broader variety of structures than infinite crystals, because they are not constrained by translational invariance on an infinite lattice. For example, the *structural landscape* of small metal particles that consist of a few tens to a few hundreds of atoms is much richer than that of their bulk material counterparts.^1−4^ Different factors cooperate at rendering this variegated scenario: first of all, possible structures are not limited to fragments of bulk crystals, but they include noncrystalline motifs, such as icosahedra or decahedra, which contain 5-fold symmetries that are forbidden in infinite crystals.^5^ Moreover, for small sizes, also planar, cage-like, and amorphous clusters have been observed,^6−8^ along with hybrid structures that exhibit features associated with more than one motif within the same cluster.^9^ Adding to this already complex scenario, metal nanoclusters are very likely to present defects, of which there are many different types. Volume defects, for instance, such as stacking faults and twin planes, are frequently observed in experiments and simulations.^10−14^ Furthermore, surface reconstructions are known to occur in several clusters,^15−18^ and internal vacancies can also be stabilized in some cases.^19,20^ Owing to the complexity of the structural landscape of nanoclusters, there is an urgent need for a robust classification method that can separate their structures into physically meaningful groups, possibly producing an informative chart of the structural landscape in terms of a small number of collective variables (CVs). In addition to providing a low-dimensional representation of the structural landscape, CVs are an essential tool to enhance sampling in configuration space, such as umbrella sampling,^21^ metadynamics,^22^ temperature-accelerated MD,^23^ and many others. A requirement of most enhanced sampling approaches is the differentiability of the chart with respect to atomic coordinates, i.e., that the CVs are differentiable functions of the coordinates.

Machine learning (ML) is emerging as an invaluable analysis tool in the field of nanoclusters, as it allows efficient navigation of the complexity of the structural landscape by extracting meaningful patterns from large collections of data. ML has already found application in microscopy image recognition,^24,25^ dimensionality reduction and exploration of potential energy surfaces,^26^ structural recognition,^26−28^ characterization of the local atomic environment,^29,30^ and machine learning force fields for metals.^31,32^

One of the main challenges in the study of nanoclusters concerns the identification of descriptors that can discriminate the various structural classes. The availability of such a tool is crucial for navigating the landscape of structures generated during simulations. In this context, the histogram of the interatomic distances, i.e., the radial distribution function (RDF), has been used to study the solid–solid transitions in metallic/bimetallic clusters via metadynamics,^33^ owing to its capability to encode structural information. Another widely used approach is Common Neighbor Analysis (CNA),^34^ a tool which relies on analyzing local atomic coordination signatures for individual atoms.^27,35^ Often, arbitrary rules^9,35^ are then applied to CNA signatures of the atoms as a means to assign the whole nanocluster to a structural family. Albeit being widely used and informative, CNA still presents certain drawbacks. First, CNA classifications are based on the arrangement of first neighbors around any given atom, and therefore, they do not directly encode information on the overall shape of the nanoparticles. In addition, even though CNA can be used for charting the structural landscape and for unsupervised clustering to obtain very refined groupings of structures (e.g., along the lines developed by Roncaglia and Ferrando^27^), the resulting chart is nondifferentiable.

In this work, we propose to use a descriptor capable of capturing in full generality the most important structural features of metal nanoclusters, the RDF, and feed it to an artificial neural network (ANN) that is trained to perform an unsupervised dimensionality reduction, yielding a low-dimensional, informative representation, where data are distributed according to their structural similarities. We start off by showing that RDFs are excellent descriptors of nanocluster structures, given their capability to describe both the local^36^ and global order together with the overall shape of diverse systems, and then we proceed to discuss the results obtained by using convolutional ANNs to reduce the dimensionality of the original descriptors.

The combination of RDF and ANNs allowed us to learn a differentiable map from the atomic positions to a low-dimensional (3D) chart of the structural features of nanoclusters of various sizes and metals. The employed data sets contain hundreds of thousands of unique structures obtained by parallel-tempering molecular dynamics (PTMD) simulations.^9,37^ It was possible to classify in an unsupervised manner this wealth of structures, reproducing the well-known CNA classes and additionally being able to distinguish subtle features present in metal nanoclusters, including the location of the twinning plane stacking faults, surface defects, central vacancies in icosahedra, and intermediate/distorted structures. The chart also allowed us to track and describe in detail dynamic structural transformations. Additional advantages of the present chart are the transferability and robustness, which was demonstrated using independent data sets of metal clusters of varying size and chemical nature, together with its differentiability (and hence suitability for CV-based exploration and biasing in molecular dynamics).

## Results and Discussion

Our goal is to gain insights into the structural complexity of metal nanoclusters by means of a differentiable map of the configuration space onto a low-dimensional yet sufficiently informative manifold (the chart).

The method consists of generating, for every cluster configuration in the data set, a set of high dimensional descriptors, the RDFs, which are known to describe both the local structural order and global shape, and distill this information representing it in a low-dimensional, highly compressed form. The specific ANN architecture we chose to perform the unsupervised dimensionality reduction is that of an autoencoder (AE)^38^ endowed with convolutional layers that renders it highly specialized at learning from numerical sequences.^39^ A dimensionality reduction step follows the convolutions, yielding a physically informed three-dimensional (3D) chart of the structural landscape of our data set, which allows us to navigate and easily understand it. Finally, we applied a clustering technique to the 3D chart to gauge its quality and to identify different structural families.

**Figure 1:**
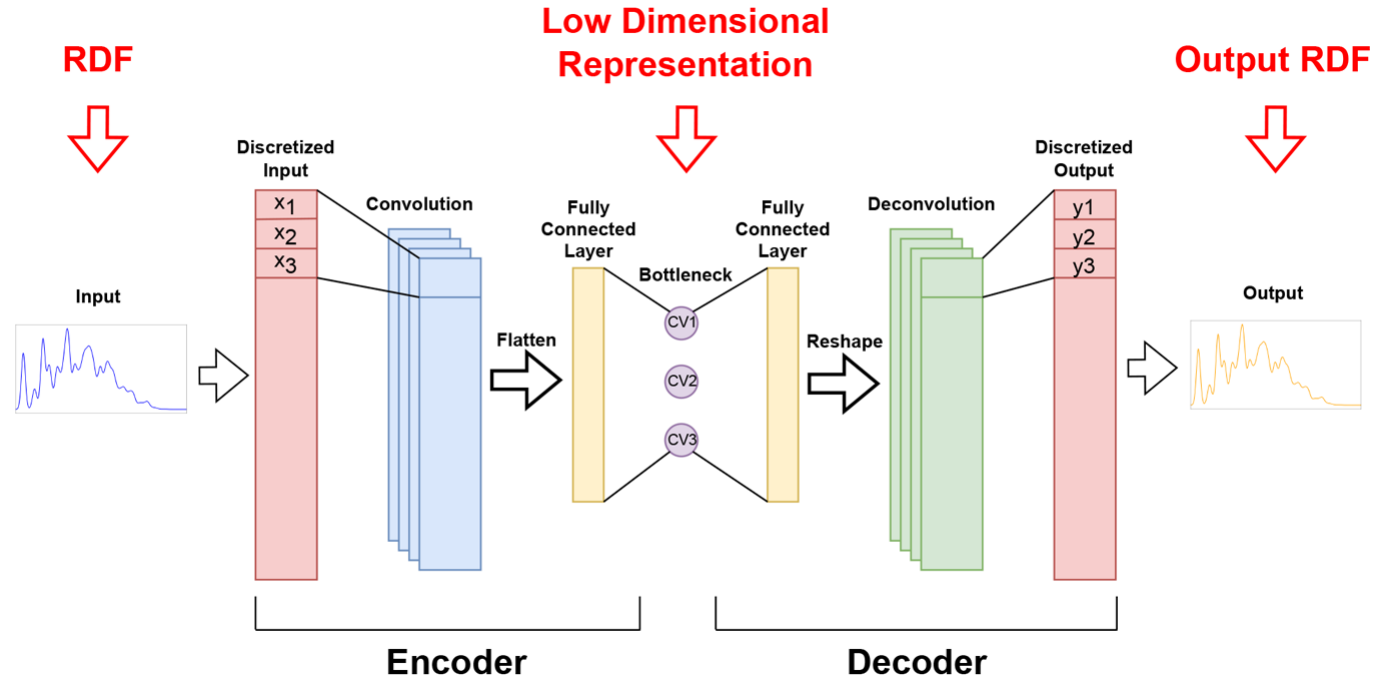
Simple sketch of the autoencoder architecture, showing how encoder and decoder meet at a low-dimensional (3D) bottleneck.

AEs constitute a particular class of ANNs that is highly specialized in the unsupervised dimensionality reduction of data.^38^ AEs are designed to reproduce the input while forcing the data through a bottleneck with a severely reduced dimensionality (Figure 1). In this way, the network needs to learn, in the first section of the network (encoder), an efficient representation of the data in such a way that the information can then be reconstructed by the second half of the newtork (decoder) with sufficient accuracy. The quality of the reconstruction is measured by a loss function that is also used in the training of the network.

Convolutional layers, which are specialized at learning from ordered sequences, are adopted in the AE hereby presented because discretized RDFs are by all means sequences. They work by applying different kernels that slide along the data, allowing the recognition of local features and patterns, which makes them well versed for the analysis of inputs like signals (using 1d convolutional kernels) or images (2d kernels). Moreover, the connections between the nodes and the related parameters are considerably reduced as compared to the fully connected layers used in standard ANN, which decreases the computational cost while allowing for better performances.

In order to test the method, we took advantage of the large data set of nanocluster structures produced by the group^9,37^ via parallel tempering molecular dynamics (PTMD) for gold, silver, and copper nanoclusters of different sizes. In the next section, we discuss in detail the results obtained for the most challenging case, a gold cluster of 90 atoms, Au_90_, while results relative to other metals and sizes will be shown in later sections.

### Structural Landscape of Au_90_

Gold nanoclusters represent an ideal test case, owing to the broad variety of structures^6−9,15,40^ they present, which include face-centered-cubic (fcc) lattice, twins, icosahedra (Ih), and decahedra (Dh). In the following, nanoclusters will be broadly classified into such standard structural families by CNA (in addition to the mix and amorphous classes), as used by Settem et al.,^9^ with the aim of having an independent benchmark for our unsupervised study. Here, we focus on a small gold nanocluster, Au_90_, which is characterized by an extremely challenging structural landscape owing to the large fraction of surface atoms. In particular, we chart a set of Au_90_ configurations extracted from PTMD simulations^9^ exploring a total of 35 temperatures ranging from 250 to 550 K. Starting from an initial set of 921 600 atom configurations, we performed a local minimization and filtered out duplicates, reducing the data set to 49 016 independent configurations.

**Figure 2:**
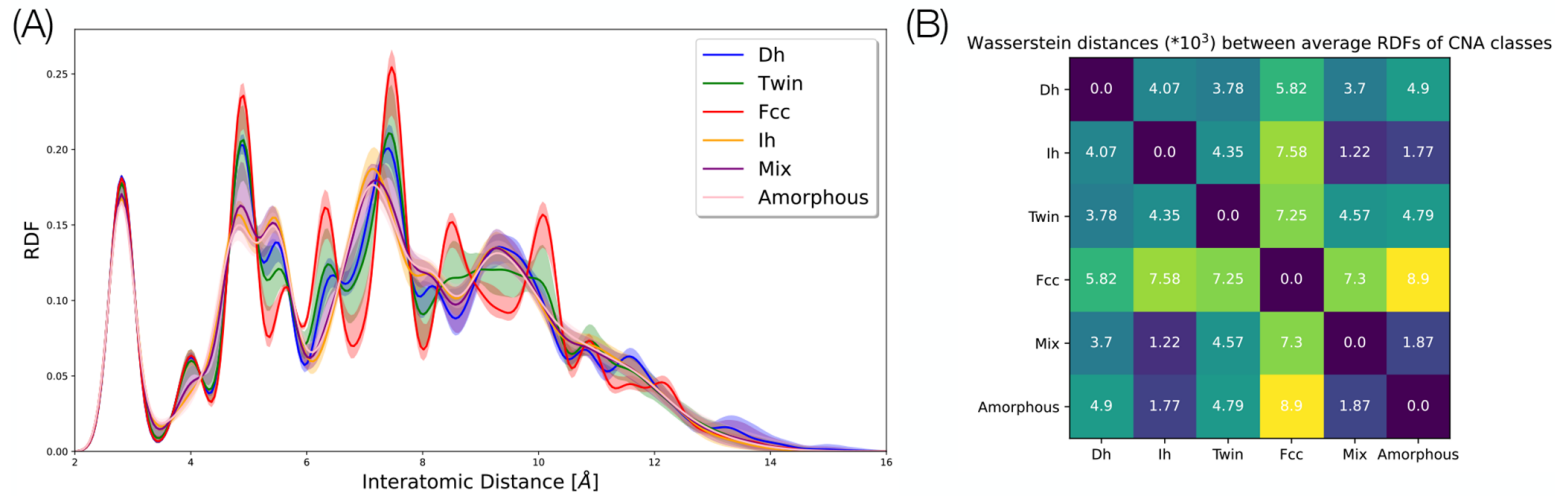
(A) Radial distribution function families for Au_90_. Colors reflect cluster structure classification provided by the CNA. Blue is used for Dh, green for twin, red for fcc, orange for Ih, purple for mix, and pink for amorphous. Shaded areas represent intervals containing 90% of the data for each CNA label, with the lower boundary representing the 0.05 quantile of the RDF population and the upper boundary the 0.95 quantile. (B) Heat map of the Wasserstein distances between the averages of the RDFs of the six CNA families is reported. Values of the distances are scaled by a factor 10^3^.

As previously mentioned, RDFs were chosen because they are general descriptors of short- and long-range order^41,42^ that are equivariant with respect to rototranslation and permutation of the atom coordinates. The aptness of RDFs as structural descriptors is well demonstrated by Figure 2, in which the RDFs of all CNA classes (fcc, twin, Dh, Ih, mix, and amorphous) are well separated. We will show in the following that this descriptive power also applies to other metals and nanocluster sizes that actually have a less rich structural landscape. However, a major drawback of using a probability distribution as a descriptor, even in its discretized version, is its high dimensionality. Our approach to provide an efficient charting of the structural landscape of metal nanoclusters, i.e., a low-dimensional representation, relies therefore on a dimensionality reduction step.

A large number of RDFs, corresponding to individual PTMD-derived structures, are used to train an autoencoder (AE), which automatically learns to compress the high-dimensional RDF information to a 3D latent representation (Figure 1). Our AE is composed of an input and an output layer, a central block, comprising the bottleneck layer, formed by three fully connected layers, while the cores of the encoder and the decoder are formed by convolutional layers (Figure 1). The training was run feeding the AE with the RDF data set (49 016 independent data), split in training and validation sets; the mean squared error (MSE) between the output and the input RDF is used as the loss function. We chose to adopt a latent space dimensionality of 3. This choice allowed for better performances in terms of the loss function as compared to higher compressions, while still allowing for a convenienent visual representation. We refer to the Supporting Information for a comparison of the results obtained by varying the dimensionality of the latent space.

**Figure 3:**
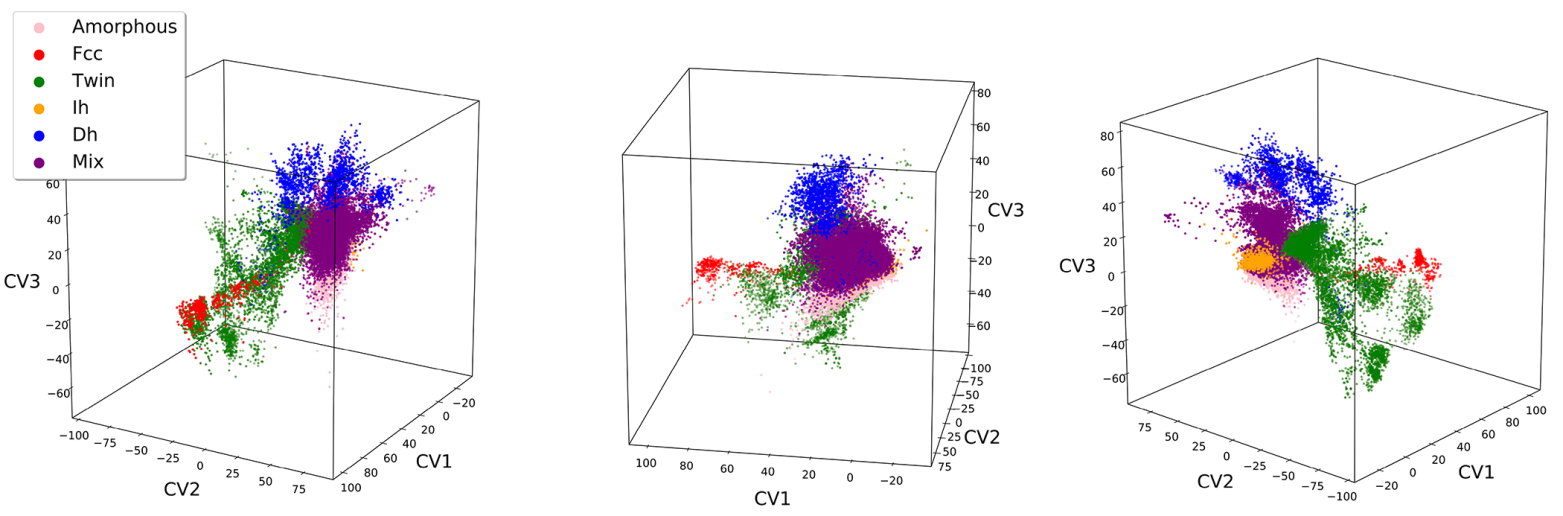
Visualization of the 3D chart generated via convolutional AE for Au_90_ data set, from different perspectives. Individual points refer to a given Au_90_ configuration in the data set mapped according to their latent space representation. The three latent coordinates are referred to as CVs. Points are colored following their (independent) CNA label classification; the color code is the same as that used in Figure 2.

The 3D chart obtained by the AE is shown in Figure 3 with data points colored by their CNA label. This representation clearly indicates how each structural family is grouped in separate regions of the chart and how their spatial ordering and distance reflect affinities among these families: similar structures are placed close together (e.g., fcc and twin), while structures that share common features occupy intermediate regions (e.g., the twin region is interposed between fcc and Dh). Overall, the obtained chart allows for a physically meaningful representation of the structures. The scatter in the data suggests that the resolution of the analysis of the chart allowed by the CNA summary labels is not fully conclusive and that further analysis can allow for a better understanding of the physical information encoded in the structure distribution inside the latent space and, consequently, a finer discrimination of different families of structures.

**Figure 4:**
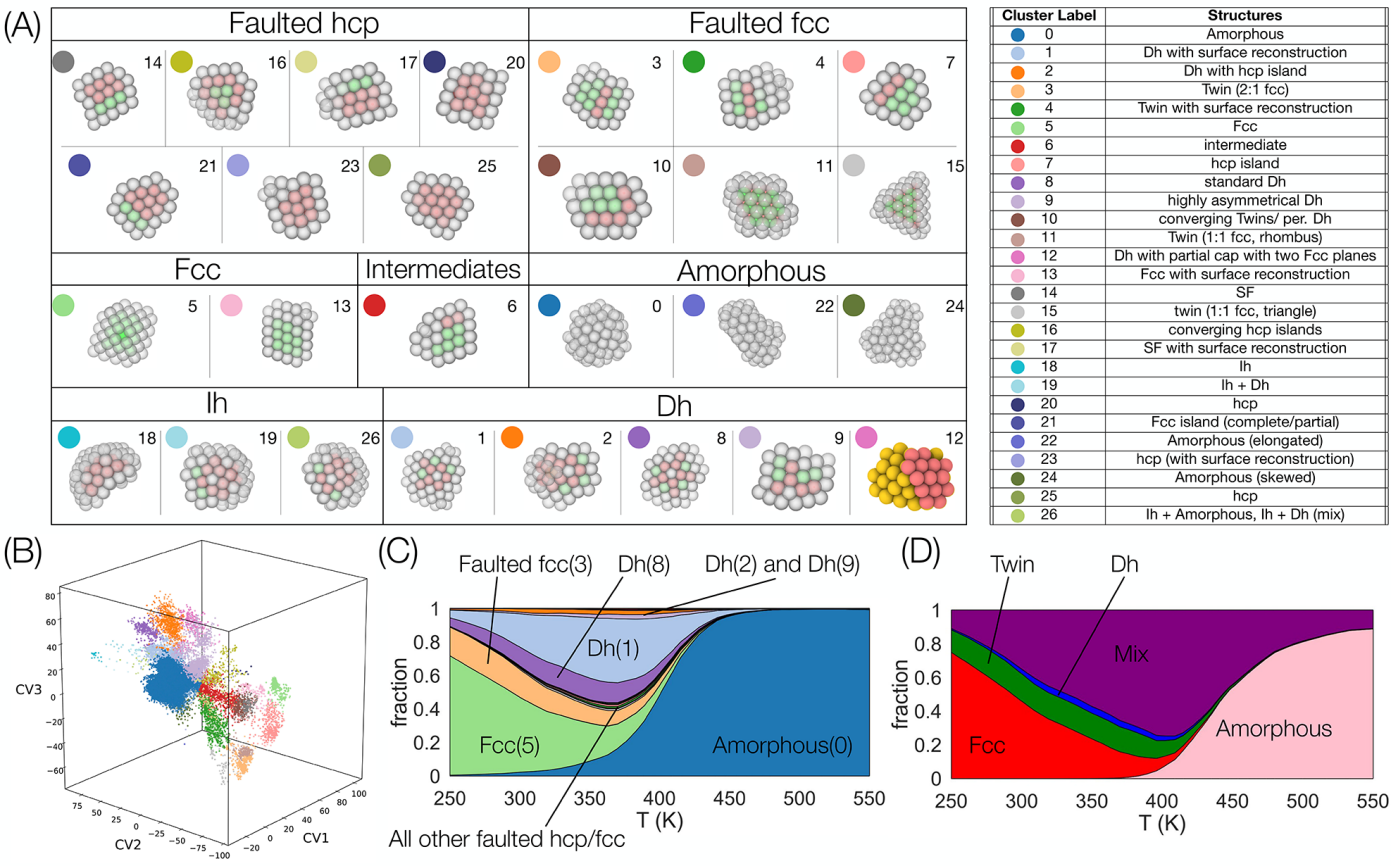
(A) Representative samples for each of the 27 structural families identified via application of the mean shift clustering algorithm on the latent space representation of the Au_90_ data set. These 27 classes were subsequently grouped in seven bigger families by similarity. Atom colors refer to their coordination: green represents atoms with fcc coordination; red stands for hcp coordination, and white for neither of the previous ones. Atomistic representations with transparency report 3D views, whereas those in solid colors represent cross sections. Every structure is given a numeric index associated with the label of the belonging cluster and a particular color. The table on the right reports both the numeric and color labels of the clusters, along with a description of the various structures. (B) Single view for a 3D plot, analogous to the one on the extreme right of Figure 3 except for the coloring, which is now representative of the labels assigned by the mean shift through the same color coding reported in panel A. (C) Mean-shift families fractions as a function of the temperature in the whole PTMD data set. The color code is the same of panels A and B. More likely structures are represented with the same name of the macrofamily, numeric index, and color of panel A. (D) Plot analogous to panel C with the only difference that the PTMD data has been classified using the CNA label classification as in the work of Settem et al.^9^ Color code and labeling are the same as those used in Figure 2.

In order to increase the structural resolution and to gain deeper insight into the physical information encoded in the latent space, we applied a clustering technique to identify meaningful and coherent regions in the chart. In particular, we chose a nonparametric technique known as mean shift.^43^ Application of this method to the 3D chart of Figure 4 was justified not only by the nonparametric nature of the clustering technique but also by its aptness at dealing with clusters of different sizes and shapes. The only input variable required by mean shift is the bandwidth, which dictates the resolution of the analysis, with the smaller bandwidths leading to more detailed parceling of the data. We chose a bandwidth that yields a robust clustering of the chart with sufficient detail, as discussed in the Supporting Information. Our analysis resulted in a robust discrimination of 27 major regions for the Au_90_ chart, corresponding to 27 different major structural families, as reported in Figure 4. From the figure, it is immediately apparent how the mean shift classification is able to distinguish and split clusters that belong to spatially separated regions of the chart, properly reflecting the ordering of the data.

Representative structures of each mean shift family are shown in Figure 4A, while Figure 4B shows the 3D chart with the points colored according to the same families. They are broadly categorized into Ih, Dh, fcc, faulted fcc, faulted hcp, intermediates, and amorphous. Faulted fcc nanoclusters are those with a predominant fcc part but which contain twin planes and stacking faults. Faulted hcp clusters are those with a predominant hcp part but which contain twin planes and/or stacking faults. Typically, structures observed in experiments and simulations are classified into basic structural families,^9,35,44−46^ which rarely capture the fine geometrical details within a given family. In contrast, our approach leads to a physically meaningful classification along with capturing the fine structural details by splitting the broader families into several subfamilies. A closer look at the various fcc and hcp faulted nanoclusters illustrates this point. There are three subfamilies (cluster 3, cluster 11, cluster 15) that contain only one hcp plane. Cluster 3, referred to as 2:1 fcc, consists of two and one fcc plane(s) on either side of the hcp plane. Similarly, clusters-11 and 15 are 1:1 fcc with differing shapes. When the hcp plane is adjacent to the surface layer, we have hcp islands (clusters-7). Cluster 10 has two converging hcp islands. In cluster 4, local surface reconstruction occurs along with a single hcp plane. Moving on to faulted hcp structures, three hcp planes converge in cluster 16. With the increase in the number of parallel hcp planes, we have either stacking faults (cluster 14) or fcc islands (cluster 21) which contain one fcc plane (opposite of the hcp island). In the extreme case, we have full hcp particles (clusters-20, 25). Clusters-17 and 23 both undergo local surface reconstruction similar to cluster 4.

In fcc families, we have the conventional fcc structures (cluster 5) and fcc structures with local surface reconstruction (cluster 13). In the case of decahedra, there are five subfamilies. Clusters 8, 9, and 12 are all conventional decahedra. In cluster 9, the decahedral axis is at the periphery, as opposed to clusters 8 and 12. Additionally, cluster 12 has a partial cap on top (atoms belonging to the cap are shown in red color). Decahedra in cluster 2 have an hcp island on the surface. Finally, decahedra also exhibit reconstruction at the reentrant grooves, resulting in icosahedron-like features (cluster 1). There are three icosahedral clusters: Cluster 18 consists of incomplete noncompact icosahedra; cluster 19 is a combination of Ih and Ih+Dh (has features of both Ih and Dh) while cluster 26 is a combination of Ih+Dh and Ih+amor (has features of both Ih and amorphous). Similarly, there are three types of amorphous structures (clusters 0, 22, and 24). Finally, we have intermediate structures in cluster 6.

The structural distributions of Au_90_, i.e., the fraction of various families as a function of temperature, of the PTMD data according to mean shift and CNA labels are shown in Figure 4C and D, respectively. In both cases, we found conventional structure families. However, mean shift further refines the CNA-based classification.^9^ For instance, with the mean shift, we have a clear separation of the various types of Dh that were previously grouped together in a broad group of mixed structures. In the case of faulted structures, there is a prominent faulted fcc cluster (Faulted fcc-3) while all other faulted structures (band between Faulted fcc-3 and Dh-8 in Figure 4C) have very low fractions. It is noteworthy that a mean shift can classify even structures that have a very low probability of occurrence.

In short, the Au_90_ analysis showcased the descriptive power of RDFs and the capability of the unsupervised dimensionality reduction performed by AE to properly compress information. Through the AE we were able to generate a highly physical representation of the data, which, rather than simply splitting different structures, is able to coherently distribute them in a 3D chart according to their physical similarities. As a consequence, the subsequent independent classification via mean shift easily identified a wealth of distinct structures and underscored the capability of the approach to distinguish both local and global structural motifs: location of twinning planes, surface defects, distorted cluster shapes, etc.

### Generality of the Approach

In this section, we show that the approach adopted for Au_90_ is of general applicability. At the root of such generality is the wealth of structural information carried by RDFs, which are expected to be valuable for a broad class of systems which includes nanoclusters of other metals and sizes, as showcased below, but is not limited to them.^3,29^

Here, we focus on larger cluster sizes that, as a general trend, show a lower variety of structures compared to smaller ones. In particular, we study clusters of 147 atoms with elemental gold (Au_147_), copper (Cu_147_), and silver (Ag_147_). These two latter cases exhibit rather different properties compared to the gold clusters; in particular, they exhibit a lower differentiation in the structural landscape that is mainly dominated by Ih structures. We discuss only selected structural families identified by the method for the three cases, that best showcase the discerning capabilities of the method: faulted structures characteristic of Au_147_ and the different types of Ih present in Ag_147_. Results for Cu_147_ are similar to those for Ag_147_ and are reported in the Supporting Information. These two examples put our approach to a test, because these two families are characterized by distinct structural features: faulted structures mainly differ for small changes in the overall shape of the particles and for their atomic coordination, while Ih have more similar shapes and lower degrees of crystallinity.

**Figure 5:**
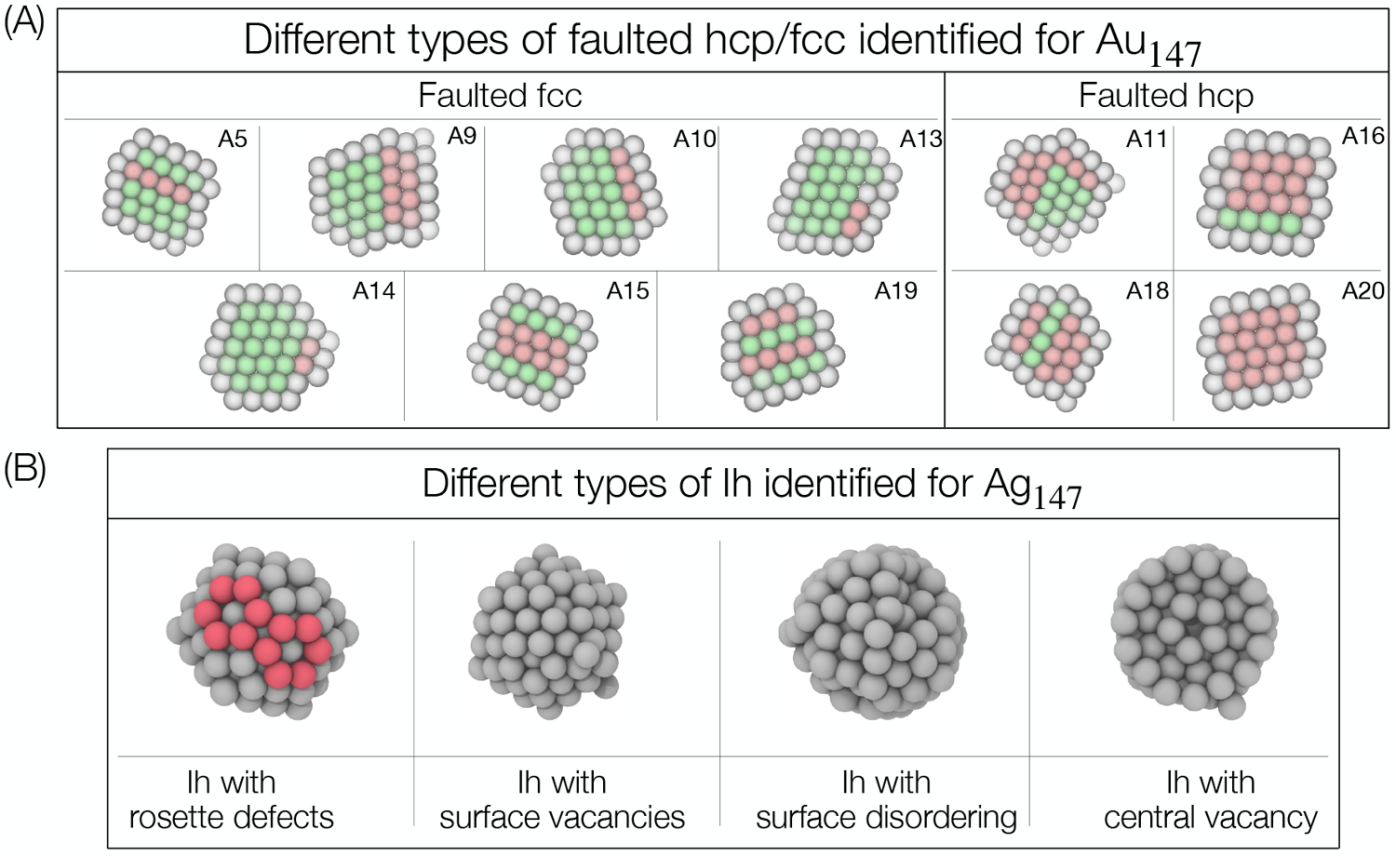
(A) Cross sections of the different types of twin families obtained by using mean shift clustering on the latent space representation of Au_147_. The families were split into two groups, in the same fashion as our treatment for the Au_90_ twin structures. Colors of the atoms refer to their individual coordination, similarly to Figure 4. Every structure is labeled with the same alphanumeric index of Figure 6A, where the 3D chart of Au_147_ is depicted. (B) The four different families of icosahedral structures for Ag_147_ are sketched. As customary, the families were extracted via mean shift clustering in the 3D space resulting from encoding of the Ag_147_ data set. The six-atom rosette defects are highlighted in red. The first three figures on the left are three-dimensional representations; the last figure is a cross section. Complete description of the clustering of all of the Ag_147_ structures can be found in the Supporting Information.

Figure 5A shows that, in the case of Au_147_, our approach is capable of distinguishing fine features in the large family of faulted structures, which are broadly grouped into faulted fcc and faulted hcp, in analogy to Au_90_. In the standard faulted fcc (A5, corresponding to a standard double twin), there is a single hcp plane with at least one fcc plane on either side. When the hcp plane is adjacent to the surface layer, we have hcp islands (A10) or sometimes partial hcp islands (A13, A14). In addition, an hcp plane and an hcp island can occur within the same structure (A19). When there is more than one hcp plane, stacking defects are observed. In the extreme case, it can be completely hcp (A20) or fcc island (A16). When there are two hcp planes, depending on the location of the hcp planes, we have either the central stacking fault (A15) or the peripheral stacking fault (A9). In the standard faulted hcp (A18), there is a single fcc plane with at least one hcp plane on either side. Finally, we have the faulted hcp cluster with converging hcp planes (A11).

Owing to the particular characteristics of silver, the structural landscape of Ag_147_ is largely dominated by icosahedra, which the clustering method is able to split into four subfamilies (Figure 5B). Conventional Ih consisting of surface vacancies is dominant among them. Icosahedra also undergo reconstruction and disorder through “rosette” defects on the surface. When the disordering increases further, we observe Ih with surface disordering. Finally, one can recognize Ih with a central vacancy where the central atom is missing as shown in the cross section in the rightmost panel of Figure 5B. Distinguishing with ease the latter structural subfamily is a feature of our approach; indeed CNA can hardly recognize icosahedra with a central vacancy because it relies on the (missing) Ih-coordinated atom to identify the Ih class.

In summary, for all of the considered cases, the method proved to be transferable and robust, being capable of characterizing the wealth of structures of Au_147_ and giving insights into the fine features distinguishing Ih subclasses for Cu_147_ and Ag_147_.

### Dynamical Structural Transitions

**Figure 6:**
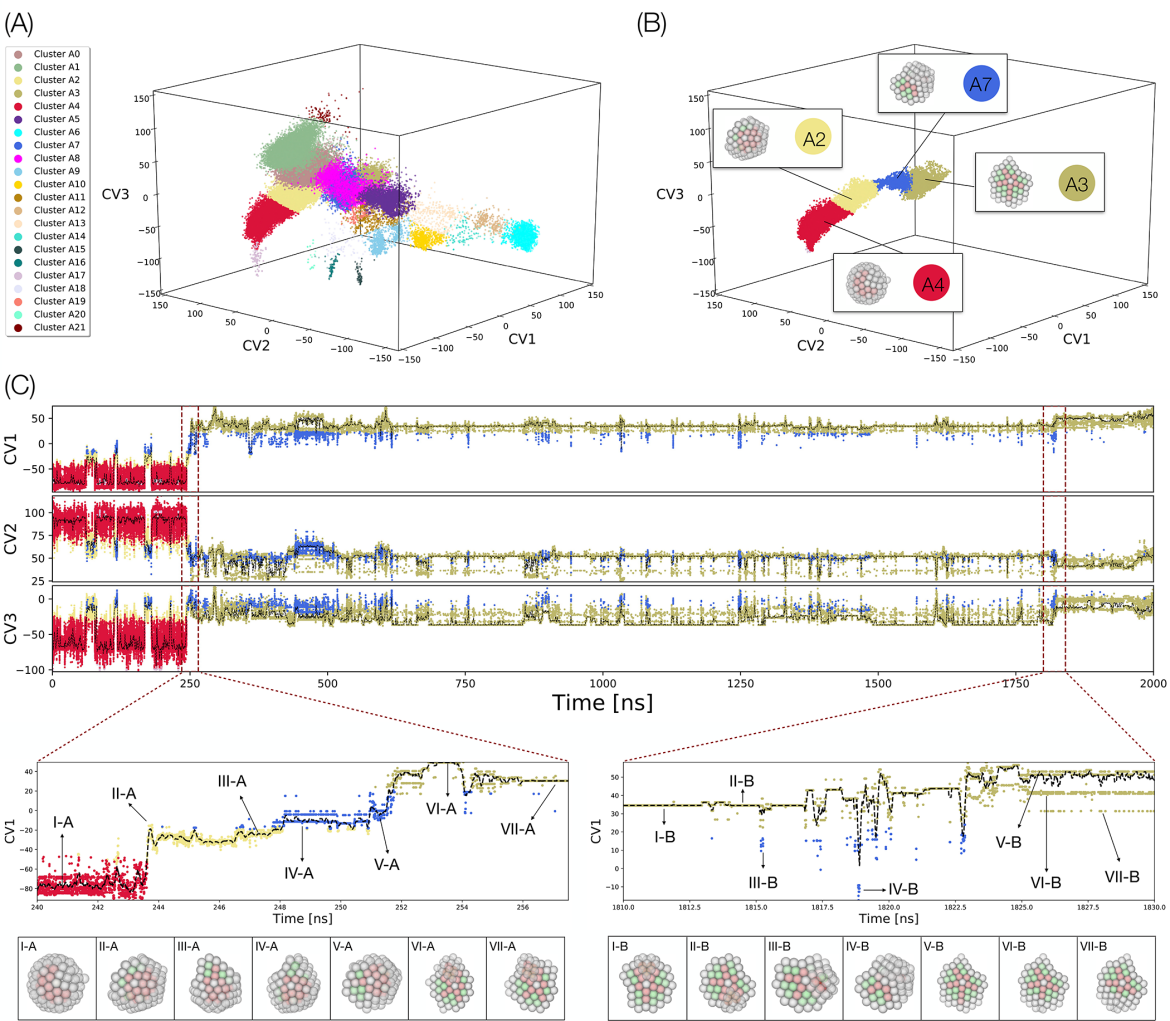
(A) Structural chart of Au_147_ containing 87 050 structures. Points are colored according to the structural families identified by mean shift clustering; see also Figure S4, now labeled using alphanumeric indexes to distinguish them by the families of Figure 4. The chart has been obtained training ex novo the network on the 87 050 of the Au_147_. (B) Plot of an unbiased MD simulation of Au_147_ undergoing a structural transition from Ih to Dh in the same chart as A. The points are colored using their mean shift classification obtained on the training data set represented in panel A. In the plot are depicted representative structures of the different regions. (C) Scatter plots of the time evolution of the three CVs along the trajectory of panel B. Dark red dashed lines highlight two intervals in which the main transformations from Ih to Dh occurs. The colors of the points correspond to their mean shift label as in panels A and B. Black dashed lines represent a running average of the scatter plots. Bottom panels report magnifications of the two main transitions with snapshots of the main structures observed.

The previous sections demonstrated that the method at hand is capable of generating reliable, low-dimensional structural charts from large data sets of nanocluster configurations for different metals and sizes. In all considered cases, the charts, informed by RDFs, excelled at distributing the different families of structures in a physically meaningful fashion, keeping similar structures closer while positioning different ones far apart. The method was able to distinguish both structures presenting major shape differences (as faulted fcc and hcp in Au nanoclusters) and structures with lower degrees of crystallinity and a closer overall shape (Ih subfamilies). In other words, the three CVs defining the chart can discriminate between different metastable states of the systems studied while maintaining an insightful ordering among them. These features suggest that the approach can be used for describing structural transitions occurring along reactive trajectories, e.g., obtained by MD simulations. To test this idea, we use the chart to study a continuous dynamic trajectory (Figure 6).

We consider a 2 μs unbiased MD run of Au_147_ at 396 K. At this temperature, the most probable structure for Au_147_ is Dh.^9^ By choosing as the initial configuration an Ih structure, which is very unlikely under such thermodynamic conditions, it is possible to observe a spontaneous Ih → Dh transition in an unbiased trajectory. In particular, we map 2 million individual MD snapshots on the chart through the AE in Figure 1, which was previously trained on independent structures generated by PTMD. To be compatible with this representation, each snapshot undergoes a short local minimization.

Figure 6A,B compares the structural chart of the entire PTMD data set with the partial representation of the same chart as obtained from the unbiased MD trajectory. The trajectory progressively populates a connected, tube-shaped region of the chart, which smoothly joins Ih to Dh domains, passing through intermediate, defected structures that belong to well-defined families. More in detail, the following structural pathway is observed: Ih (cluster 4) → distorted-Ih (cluster 2) → distorted-Dh (cluster 7) → Dh (cluster 3), which is confirmed by analyzing the structures along the trajectory (Figure 6C). Beginning from Ih there is an initial transition to distorted-Ih where the disorder increases, and we start observing fcc-coordinated atoms in the nanocluster. The distorted-Ih then changes to distorted-Dh where the amount of fcc coordinated atoms increases further. Apart from the difference in the amount of fcc, distorted-Ih is geometrically similar to Ih, while distorted-Dh is closer to Dh. Finally, the distorted-Dh transitions to Dh which completes a gradual change from Ih to Dh with physically meaningful changes along the tube-shaped region.

In the absence of the chart, it would, in principle, be possible to perform a visual analysis of the Ih → Dh trajectory of roughly 2 million structures. However, it would be extremely cumbersome to identify the main thermally activated transformation and to track the fine structural changes and fluctuations along the trajectory which are crucial for understanding the transition mechanisms. This difficulty is easily overcome by tracking changes in the chart coordinates as reported in Figure 6C, which shows the time evolution of the CVs as a function of time along the trajectory. Changes in CVs are found to correlate very well with structural changes. Three broad phases can then be distinguished during the evolution of the trajectory. In the initial phase (up to ∼250 ns), the nanocluster is predominantly Ih (cluster 4) with intermittent fluctuations to distorted-Ih (cluster 2) and distorted-Dh (cluster 7). The actual Ih → Dh transition occurs around ∼245 ns, followed by a long intermediate phase (spanning ∼245 to ∼1820 ns), in which fluctuations between Dh (cluster 3, dominant) and distorted-Dh (cluster 7, minor) are observed. A final transition step at ∼1820 ns leads to the final phase consisting of Dh with very few fluctuations to distorted-Dh. Here, we stress that this information can be obtained simply by following the CVs even before looking at the structures.

We now focus on the transition regions and look closely at the structural changes. For this purpose, we consider CV1. In the tubelike region, a continuous increase in CV1 is synonymous with a continuous change from Ih to Dh. A zoomed plot of the first transition (between 240 and 260 ns) is shown in the lower left panel of Figure 6C, see Figure S7 for CV2 and CV3. The initial Ih structures (I-A) transition to distorted-Ih structures (II-A, III-A) where we begin to see the fcc-coordinated atoms along with Dh-like features. With a further increase in CV1, there is a gradual change to distorted-Dh structures (IV-A, V-A). Finally, these structures transition to Dh structures that have an hcp island (VI-A, VII-A). Decahedra with an hcp island dominate the middle phase and hcp-island-free Decahedra are obtained after a final transition around ∼1822 ns (shown in the lower right section of Figure 6C). This second transition is marked by a slight increase in the mean CV1 value (black dashed line): initially, we have Dh with an hcp island (I–B, II–B) which transitions to a better Dh (without an hcp island) around ∼1823 ns (V–B). It appears that this transition is aided by fluctuations to distorted-Dh intermediates (III–B, IV–B). After the transition to a better Dh (beyond ∼1825 ns), there are three distinct horizontal branches. The dominant one, which has the highest CV1 value, corresponds to perfect defect-free Dh (V–B). However, this structure often undergoes two types of local reconstructions near the reentrant groove (VI–B and VII–B), which coincide with two distinct values of CV1.

The preceding discussion underscores that the three deep CVs are capable of describing in a detailed and physical fashion what happens during a dynamic transition. The chart enables on-the-fly tracking of the system along its structural changes and describes transitions between different metastable states. This is further evidence of the physical insightfulness of the latent space generated starting from the RDFs, underscoring the reliability of the structural information contained in the charts and further showcasing the power of the approach. In particular, the method shows promise for characterizing and analyzing long trajectories generated via molecular simulations, enabling a fast and informed way to study and follow the time evolution of this type of systems. Importantly, the differentiability of the coordinates of the latent space with respect to the atomic positions makes it possible to address the challenge of biasing MD simulations of structural changes.^33,47^ The specific merit of this approach is to provide a natural route to devise a general, informative, and low-dimensional collective variable space capable of describing dozens of structural motifs. We plan to investigate structural transformation driven by deep learned collective variables in a separate communication.

## Conclusions

This work presents an original machine learning method capable of charting the structural landscape of nanoparticles according to their radial distribution function. The approach comprises two subsequent information extraction steps. The first consists of translating the atomic coordinates into RDFs, which encode information about the structure in translationally, rotationally, and permutationally invariant ways. The high-dimensional information contained in the RDF is then reduced to a low-dimensional (3D) and yet visually insightful representation (“chart”) by exploiting convolutional autoencoders. These deep-learning collective variables are surprisingly good at describing structural features in a physically meaningful way, discriminating the different states of the system.

The 3D charts of different metal nanoclusters were then analyzed using a nonparametric clustering technique, which allowed us to classify the data points into structural families. The method succeeded at disentangling the complex structural motifs of nanoclusters having different shapes and metals (Au_90_, Au_147_, Ag_147_, and Cu_147_), distinguishing also fine differences between faulted and mixed structures as well as small defects (icosahedra with central vacancy, surface defects, etc.). Related structural motifs, e.g., fcc and faulted fcc/hcp, were found to occupy close regions of the chart, allowing us to garner insights also into dynamical structural transformations.

Finally, the method further proved to be useful in the analysis of a long unbiased MD run of Au_147_ undergoing a structural transition. The collective variables allowed us to accurately track and describe structural changes along the dynamics. This pushes the applicability of the method beyond the simple analysis of structural differences in large data sets, making it a powerful tool for the inspection, interpretation, and possibly generation of reactive trajectories between metastable states. Indeed, the ability to discriminate with a high level of detail different metastable states, together with the intrinsic differentiability of neural networks, makes the encoded variables promising for low-dimensional CVs for biased MD simulations.

The excellent results obtained for metal nanoclusters, for which the method could learn to identify a variety of structures ranging from crystalline to faulted and amorphous, demonstrate the virtue of machine learning on radial distribution functions. Building on the generality of its descriptors, this machine learning framework could be used to chart the structural landscape of diverse kinds of systems including nonmetallic nanoparticles^28,48^ and colloidal assemblies,^29,49,50^ advancing our capability to classify, explore, and understand transitions in these systems.

## Methods

The original data sets we considered included hundreds of thousands of structures for each particular cluster size and type. The structures were generated through parallel-tempering molecular dynamics (PTMD) simulations (see Supporting Information). For every data set, original structures were then locally minimized to discount thermal noise. In order to avoid redundancy in the data, due to duplicates in the locally minimized structures, the initial set of structures was filtered out in order to select only unique samples. This selection was based on both CNA classification and potential energy. As a result, structures in the final data set differed from each other by at least 0.1 meV in the potential energy or by CNA label, leading to a reduction in the number of structures to a few tens of thousands for every cluster type. The RDFs of each configurations were obtained using kernel density estimation on the interatomic distances (using the KernelDensity library from scikit-learn package^51^) with Gaussian kernels and a bandwidth of 0.2 nm.

The RDFs were then discretized and processed by the autoencoder, as described in Figure 1. Input and output of the AE share the same sizes, equal to the total mesh points of the discretized RDFs. The convolutional part of the encoder is composed of five blocks made of a convolutional layer, a rectified linear unit activation function, and a batch normalization. After the convolutions, the outputs were flattened and fed to a fully connected linear layer which outputs the three CVs values, closing the encoder section. The decoder follows, mirroring the encoder. The three outputs of the encoder were fed to another fully connected layer whose output is reshaped and fed to five deconvolutional blocks that replicated, mirrored, the convolutional part of the encoder. Finally, in the output layer of the decoder, data returned to their initial size.

The output was compared to the input in the training using the MSE loss. We performed an independent training for every nanocluster composition and size. More details regarding the AE architecture parameters and the training can be found in the Supporting Information. After the training, the three-dimensional output of the bottleneck was evaluated for all of the data to obtain a 3D chart, e.g., the one reported in Figure 3. After the chart of the data has been generated, the mean shift^43^ clustering technique was exploited to identify families of structures and evaluate the quality of the chart. Mean shift requires setting only one parameter, the bandwidth, dictating the resolution of the analysis. Bandwidth selection was obtained looking for intervals of values, yielding an (almost) constant number of clusters, see Figure S3.

Finally, the 50 configurations closest to each centroid were analyzed visually, in order to inspect for major structural features characterizing the different regions identified by the clustering.

## Data Availability

Additional data are available at 10.5281/zenodo.10018329.

## Supporting Information Available

PTMD data set composition; structure of the autoencoder; training parameters; bandwidth selection for mean shift; complete charts and clustering results for Au_147_, Ag_147_, Cu_147_; plots of the CVs during main transitions in the dynamical trajectory (PDF)

## Notes

The present manuscript was released as a preprint arXiv under the identifier arXiv.2306.12874. Emanuele Telari; Antonio Tinti; Manoj Settem; Luca Maragliano; Riccardo Ferrando; Alberto Giacomello. Charting nanocluster structures via convolutional neural networks. 2023, 2306.12874. arXiv. 10.48550/arXiv.2306.12874 (accessed October 11th, 2023).
