# Supplementary material for: Charting Nanocluster Structures via Convolutional Neural Networks
Source: ACS Nano. 2023 Oct 19;17(21):21287–96. doi: 10.1021/acsnano.3c05653 (PMC10655179; doi:10.1021/acsnano.3c05653)
Supplement: Supplementary file 1 — nn3c05653_si_001.pdf [file nn3c05653_si_001.pdf]

Supporting Information Charting nanocluster structures via convolutional neural networks

Emanuele Telari,[†] Antonio Tinti,[*,†] Manoj Settem,[†] Luca Maragliano,[‡,¶] Riccardo Ferrando,[*,§] and Alberto Giacomello[*,†]

†*Dipartimento di Ingegneria Meccanica e Aerospaziale, Sapienza Università di Roma, Rome 00184, Italy*

‡*Dipartimento Scienze della Vita e dell'Ambiente, Università Politecnica delle Marche, Ancona 60131, Italy*

¶*Center for Synaptic Neuroscience and Technology, Istituto Italiano di Tecnologia, Genova 16132, Italy*

§*Dipartimento di Fisica, Università di Genova, Genova 16146, Italy*

E-mail: antonio.tinti@uniroma1.it; ferrando@fisica.unige.it; alberto.giacomello@uniroma1.it
Phone: +39 06 44585200

Datasets

Filtering of the structures

To avoid duplicates of structures that could bias the training of the network and the following clustering, we filtered the PTMD datasets, such that every structures differed from the other for at least 0.1 meV in potential energy or for CNA classification. In the following sections are listed the datasets compositions before and after the filtering, following the CNA

classification of the structures.

Au_{90} dataset composition

```
PTMD data

Dh data = 15,520
Ih data = 789
Twin data = 83,471
Fcc data = 142,286
Mix data = 406,164
Amorphous data = 273,370

Total = 921,600
```

```
Filtered data

Unique Dh = 1,431
Unique Ih = 766
Unique Twin = 5,946
Unique Fcc = 495
Unique Mix = 19,946
Unique Amorphous= 20,432

Total = 49,016
```

Au_{147} dataset composition

```
PTMD data
```

Dh configurations = 278,405

Ih configurations = 28,911

Twin configurations = 29,553

Fcc configurations = 19,248

Mix configurations = 69,641

Amorphous configurations = 188,114

Total = 613,872

Filtered data

Dh = 11,873

Ih = 10,935

Twin = 8,839

Fcc = 2,689

Mix = 26,615

Amorphous = 26,099

Total = 87,050

Ag_{147} dataset composition

PTMD data

Dh configurations = 1,170

Ih configurations = 117,844

Twin configurations = 671

Fcc configurations = 1

Mix configurations = 5,871

Amorphous configurations = 53,643

Total = 179,200

Filtered data

Dh = 712

Ih = 3,065

Twin = 614

Fcc = 1

Mix = 5,249

Amorphous = 20,764

Total = 30,405

Cu_{147} dataset composition

PTMD data

Dh configurations = 579

Ih configurations = 180,260

Twin configurations = 734

Fcc configurations = 1

Mix configurations = 6,422

Amorphous configurations = 36,004

```
Total = 180,260
```

```
Filtered data

Dh = 467
Ih = 2,409
Twin = 691
Fcc = 1
Mix = 5,670
Amorphous = 18,619

Total = 27,857
```

Autoencoder

Inputs

The input of the AE are sequences of values representing the discretization RDFs of the structures composing the datasets. These have been obtained using kernel density estimation (using KernelDensity library from scikit-learn package[1]) on the interatomic distances of the single structure in order to obtain a smoothed histogram. The kde have been applied using gaussian kernels and a bandwidth of 0.2. The kde have been then fitted on the distances ranging from 0 to 25 angstrom, discretized in 500 bins, for all the metal nanoclusters studied. Before feeding the RDFs to the CNN autoencoder, they have been subsequently cleaned from the points where their value was identically zero for all the data in the dataset, corresponding to highest or very low distances. The final form of the input descriptors is then a kernel density estimation of the interatomic distances discretized in 340 points, ranging from 2.004 to 18.988 angstrom for Au_{147}, Ag_{147}, while for Au_{90} and Cu_{147}, due to the smaller sizes of

the particles, only 280 points have been fed to the CNN, ranging from ranging from 2.004 to 15.982 angstrom. In Fig. S1 in the different panels are plotted the RDFs for all the clusters we analyzed. All the dataset have been fed to the autoencoders splitting them in training and validation set, following the same proportion: 80% of the data composed the training set and 20% the validation set. The training set has been then divided in batches of 128 elements.

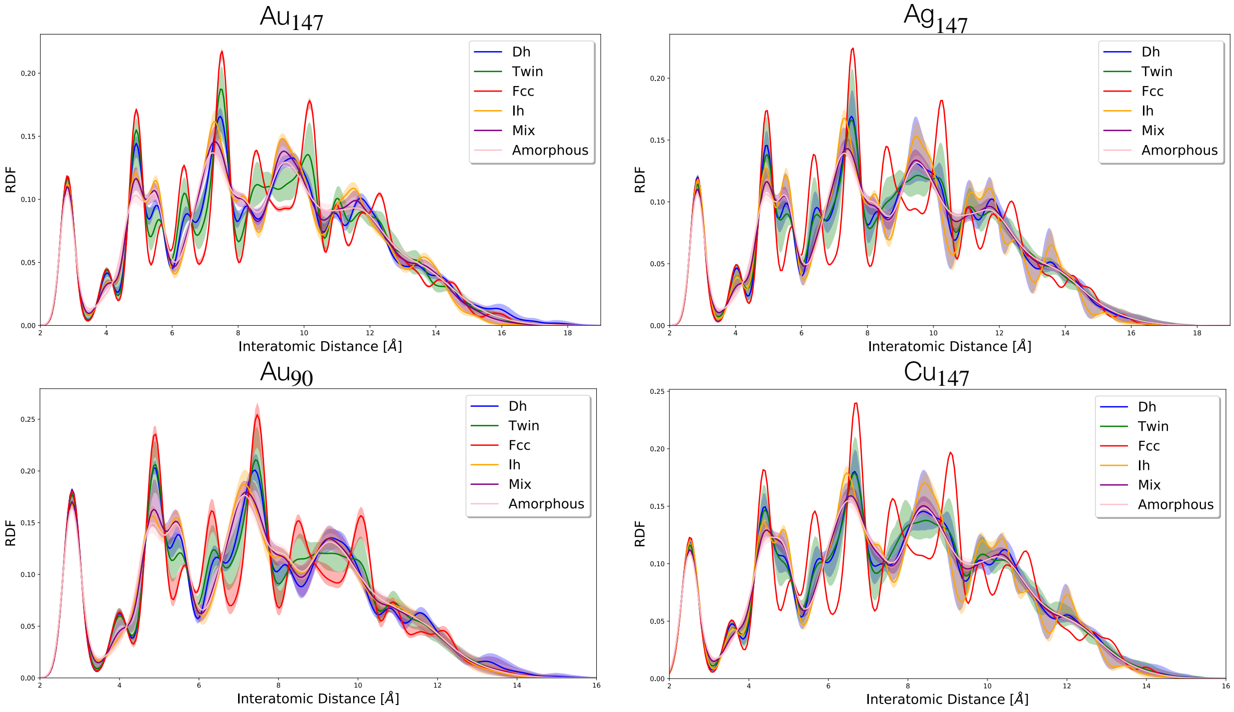

Figure S1: Plots for all the 4 metal clusters studied of the RDFs used as input to the AE, grouped following the CNA classification. Shaded regions represent the scattering of the data included between the 0.95 and 0.05 quantiles.

Dimensionality of the latent space

In order to choose the dimensionality of charts, i.e. the size of the bottleneck layer of the AE, we measured, in terms of loss, the performance of the AE while varying the the bottleneck size and keeping all the other parameters (related to AE structure and training) fixed. The best values for the loss achieved for the particular case of Au_{147} after 100 epochs of training are reported in Fig. S2

The performances, as showed in the plot, predictably tend to improve when increasing the

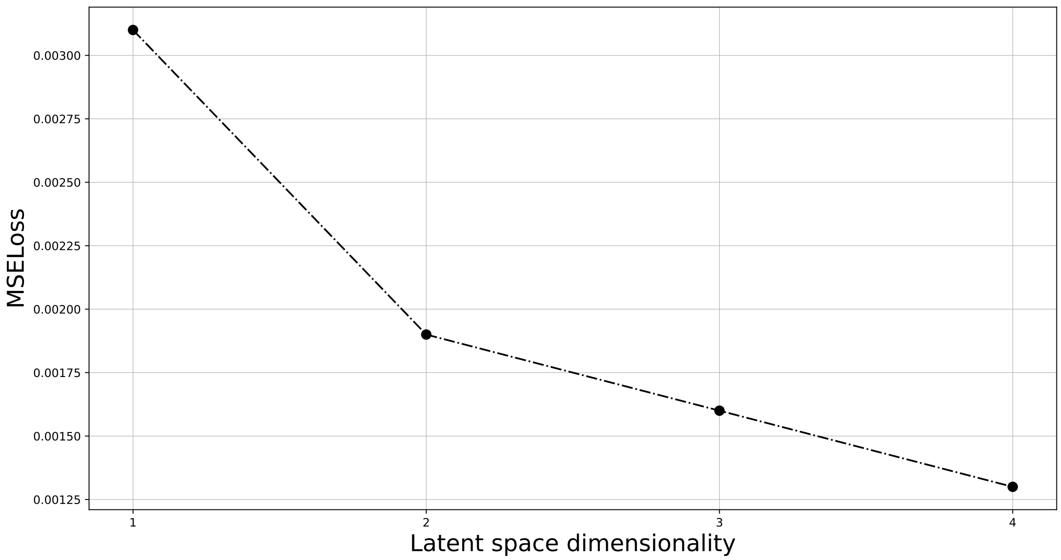

Figure S2: Plots of the MSELoss achieved after 100 epochs of training for the Au_{147} dataset. All the training where conduced changing only the bottleneck size of the AE, and letting untouched all the other parameters regarding the AE structure and the training. We chose a bottleneck size, i.e the latent space dimensionality, equal to 3, in order to achieve the best performances in the training while having a visualizable and easier to analyze chart.

dimensionality of the latent space. An elbow is found when increasing from 1 to 2 latent space dimensions indicating that two latent coordinates can already provide a fair charting of the structural space. In the end we chose to pick a dimensionality of 3, which allowed for better charting performances while still allowing for a convenient visualization and analysis of the latent space. The choice was supported by the good results obtained *a posteriori* when performing detailed analysis of the structures resulting from clustering on the latent space.

We attach here, in Fig. S3, also the comparison between the 2D and 3D representations generated by the autoencoder for the cluster Au_{90}.

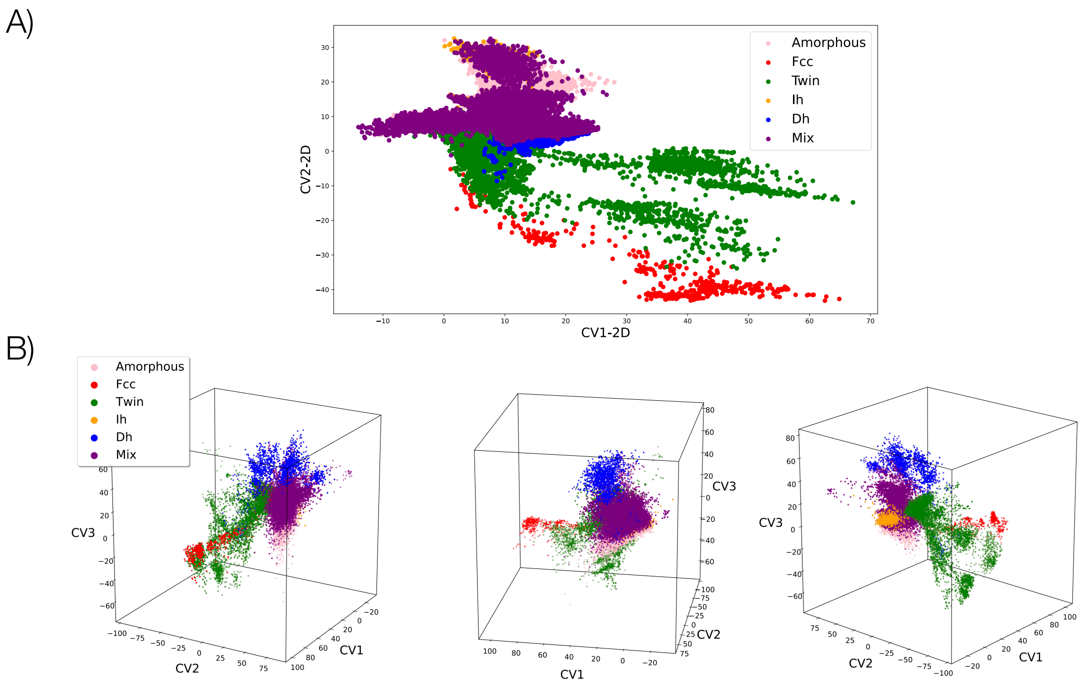

Figure S3: Scatter plot of the Au_{90} structures on the 2-dimensional space generated via the autoencoder. The autoencoder has the same exact structure of the one used to generate the 3D chart differing only for the bottleneck size. Dimensions are called as "CV-2D" to distinguish them from the one of the three chart, being obtained with a different training. B) The 3D chart of Au_{90} same of fig. 4B of the main text is reported to allow for easier comparison

Structure of the autoencoder

As described in the Methods Section of the paper, the autoencoder is composed by two main blocks, the encoder and the decoder composed by 5 convolutional layers, and a central block composed by fully connected layers. Input and output layer share the same structures and are two convolutional layers.

The convolutional layers of the encoder share the same padding, set to 0, and same stride, set to 1. The input layer is a convolution channel with 1 input channel and 64 output channels. The number of input and output channels is the same for the next three layers, equal to 64, while the last two layers give as output a decreasing number of channels, respectively 32 and 16, while the input number of channels is set to be equal to the output of the previous layer, respectively 64 and 32. The kernels have a decreasing size, being respectively of size 20,20,20,10,8,5. All convolutional layers are followed by a ReLU activation function and by a layer of batch normalization. The outputs of the final 16 channels, reaching the central block, are flattened and then passed to a fully connected layer which results in the bottleneck, the central layer composed by three nodes, with no activation function interposed. The bottleneck is followed by a fully-connected layer of the same size of the one preceding it, then the output is reshaped and passed to the decoder, which has a completely mirrored structure respect to the encoder, with the difference that the layers apply a deconvolution instead of a convolution, in order to bring back the data to their original size. Here is reported the summary of the AE used for Au_{90} as printed by torchsummary:

Listing 1: Summary of the network printed with torchsummary

```
##### [NN Architecture] #####


Autoencoder(
  (input_layer): Conv1d(1, 64, kernel_size=(20,), stride=(1,))
  (relu_1): ReLU()
  (batchnorm): BatchNorm1d(64, eps=1e-05, momentum=0.1, affine=True,
```

```
    track_running_stats=True)
  (encoder): Sequential(
    (0): Conv1d(64, 64, kernel_size=(20,), stride=(1,))
    (1): ReLU()
    (2): BatchNorm1d(64, eps=1e-05, momentum=0.1, affine=True,
      track_running_stats=True)
    (3): Conv1d(64, 64, kernel_size=(20,), stride=(1,))
    (4): ReLU()
    (5): BatchNorm1d(64, eps=1e-05, momentum=0.1, affine=True,
      track_running_stats=True)
    (6): Conv1d(64, 64, kernel_size=(10,), stride=(1,))
    (7): ReLU()
    (8): BatchNorm1d(64, eps=1e-05, momentum=0.1, affine=True,
      track_running_stats=True)
    (9): Conv1d(64, 32, kernel_size=(8,), stride=(1,))
    (10): ReLU()
    (11): BatchNorm1d(32, eps=1e-05, momentum=0.1, affine=True,
      track_running_stats=True)
    (12): Conv1d(32, 16, kernel_size=(5,), stride=(1,))
    (13): ReLU()
    (14): BatchNorm1d(16, eps=1e-05, momentum=0.1, affine=True,
      track_running_stats=True)
  )
  (maxpooling): MaxPool1d(kernel_size=2, stride=2, padding=0, dilation=1,
    ceil_mode=False)
  (flatten): Flatten()
  (embed_linear): Linear(in_features=3248, out_features=3, bias=True)
  (decode_linear): Linear(in_features=3, out_features=3248, bias=True)
  (decoder): Sequential(
```

```
    (0): ConvTranspose1d(16, 32, kernel_size=(5,), stride=(1,))
    (1): ReLU()
    (2): BatchNorm1d(32, eps=1e-05, momentum=0.1, affine=True,
       track_running_stats=True)
    (3): ConvTranspose1d(32, 64, kernel_size=(8,), stride=(1,))
    (4): ReLU()
    (5): BatchNorm1d(64, eps=1e-05, momentum=0.1, affine=True,
       track_running_stats=True)
    (6): ConvTranspose1d(64, 64, kernel_size=(10,), stride=(1,))
    (7): ReLU()
    (8): BatchNorm1d(64, eps=1e-05, momentum=0.1, affine=True,
       track_running_stats=True)
    (9): ConvTranspose1d(64, 64, kernel_size=(20,), stride=(1,))
    (10): ReLU()
    (11): BatchNorm1d(64, eps=1e-05, momentum=0.1, affine=True,
       track_running_stats=True)
    (12): ConvTranspose1d(64, 64, kernel_size=(20,), stride=(1,))
    (13): ReLU()
    (14): BatchNorm1d(64, eps=1e-05, momentum=0.1, affine=True,
       track_running_stats=True)
  )
  (output_layer): ConvTranspose1d(64, 1, kernel_size=(20,), stride=(1,))
)
----------------------------------------------------------------
        Layer (type)               Output Shape         Param #
================================================================
            Conv1d-1              [-1, 64, 261]           1,344
              ReLU-2              [-1, 64, 261]               0
       BatchNorm1d-3              [-1, 64, 261]             128
```

Conv1d-4	[-1, 64, 242]	81,984
ReLU-5	[-1, 64, 242]	0
BatchNorm1d-6	[-1, 64, 242]	128
Conv1d-7	[-1, 64, 223]	81,984
ReLU-8	[-1, 64, 223]	0
BatchNorm1d-9	[-1, 64, 223]	128
Conv1d-10	[-1, 64, 214]	41,024
ReLU-11	[-1, 64, 214]	0
BatchNorm1d-12	[-1, 64, 214]	128
Conv1d-13	[-1, 32, 207]	16,416
ReLU-14	[-1, 32, 207]	0
BatchNorm1d-15	[-1, 32, 207]	64
Conv1d-16	[-1, 16, 203]	2,576
ReLU-17	[-1, 16, 203]	0
BatchNorm1d-18	[-1, 16, 203]	32
Flatten-19	[-1, 3248]	0
Linear-20	[-1, 3]	9,747
Linear-21	[-1, 3248]	12,992
ConvTranspose1d-22	[-1, 32, 207]	2,592
ReLU-23	[-1, 32, 207]	0
BatchNorm1d-24	[-1, 32, 207]	64
ConvTranspose1d-25	[-1, 64, 214]	16,448
ReLU-26	[-1, 64, 214]	0
BatchNorm1d-27	[-1, 64, 214]	128
ConvTranspose1d-28	[-1, 64, 223]	41,024
ReLU-29	[-1, 64, 223]	0
BatchNorm1d-30	[-1, 64, 223]	128
ConvTranspose1d-31	[-1, 64, 242]	81,984
ReLU-32	[-1, 64, 242]	0

```
     BatchNorm1d-33           [-1, 64, 242]             128
  ConvTranspose1d-34          [-1, 64, 261]          81,984
           ReLU-35            [-1, 64, 261]               0
     BatchNorm1d-36           [-1, 64, 261]             128
  ConvTranspose1d-37           [-1, 1, 280]           1,281
================================================================
Total params: 474,564
Trainable params: 474,564
Non-trainable params: 0
----------------------------------------------------------------
Input size (MB): 0.00
Forward/backward pass size (MB): 3.18
Params size (MB): 1.81
Estimated Total Size (MB): 4.99
----------------------------------------------------------------
```

Training

The autoencoder has been trained using MSE loss function and Adam optimizer.[2] The starting learning rate has been set to 0.001 and then updated using a step scheduler halving its value at epoch number 30 and 90. The training was interrupted when the loss started to reach a plateau and when the obtained charts started to show no differences. All the autoencoders showed a convergence in the loss value and in the generated chart after about 100 epochs. The model showing the best loss on validation set was saved during the training. In the table below are listed the best values for the loss for the 4 studied metal nanoclusters.

	Best loss (MSE)
Au_{90}	0.0029
Au_{147}	0.0016
Ag_{147}	0.0022
Cu_{147}	0.0023

Clustering (Mean shift)

Bandwidth selection

The study of the reduced three-dimensional space was conduced using clustering techniques in order to locate interesting regions representing different categories of structures. Since the data and the structure of the reduced space were inherently highly inhomogeneous, we decided to exploit a technique suitable for clustering data in different sized and shaped cluster. We chose to use mean shift.[3] This technique requires only to set a single parameter which is the bandwidth, related to the resolution of the analysis of the space. Different techniques can be used to select the proper bandwidth.[3] Selection of the bandwidth was performed optimizing stability of the decomposition and thus minimizing the variation in the number of clusters that is obtained when gently perturbing the chosen bandwidth value (Fig. S4).

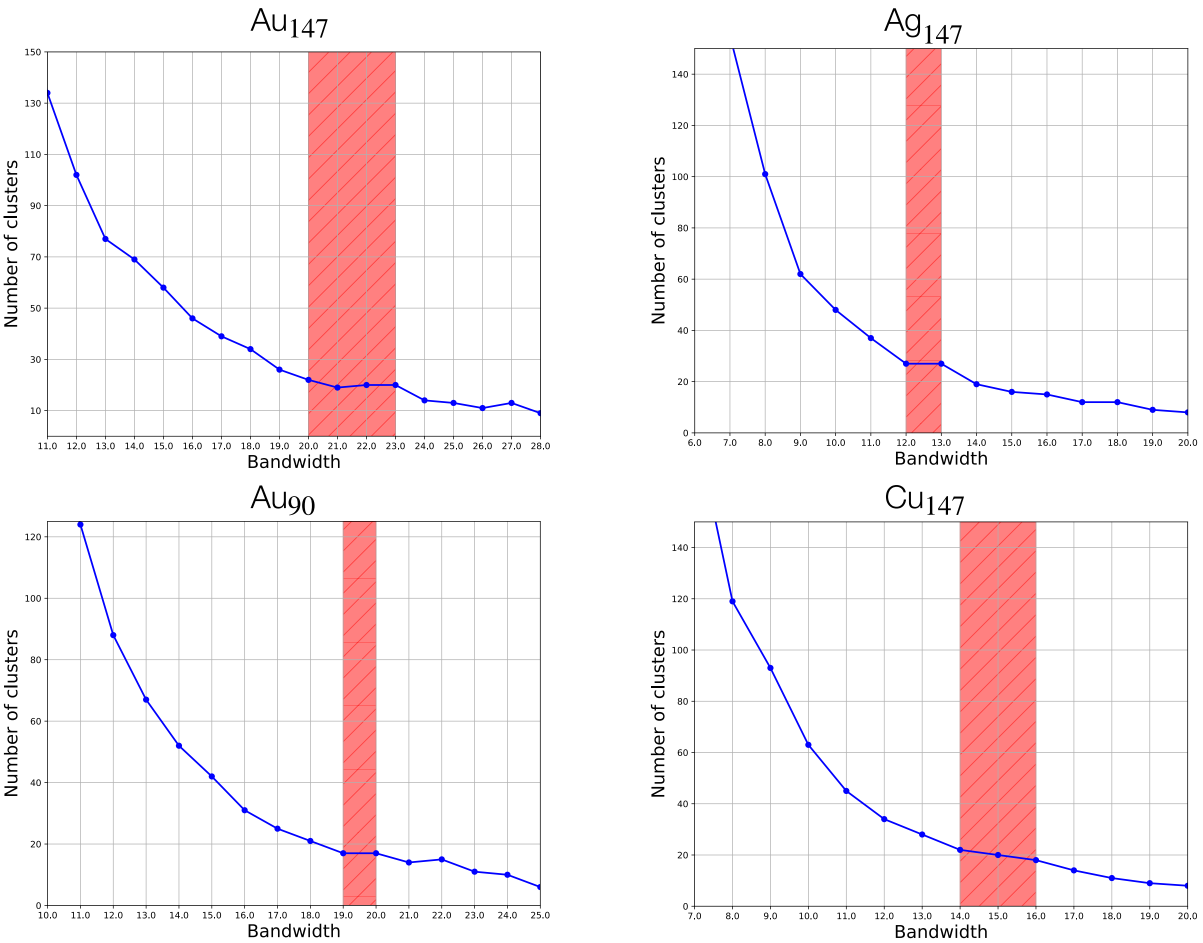

Figure S4: Plots of number of clusters as function of the MS bandwidth. The red shaded region correspond to the range of bandwidth values were the number of clusters of the decomposition shows very low dependency on the bandwidth value, indicating a good stability of the decomposition.

Results for other metallic nanoclusters

In the following subsections are reported figures with the complete charting for the other clusters studied, apart from Au_{90} presented in the main text.

Au_{147}

Chart for Au_{147} is reported in Fig. S5 with a detailed description of the families of structures identified using mean shift.

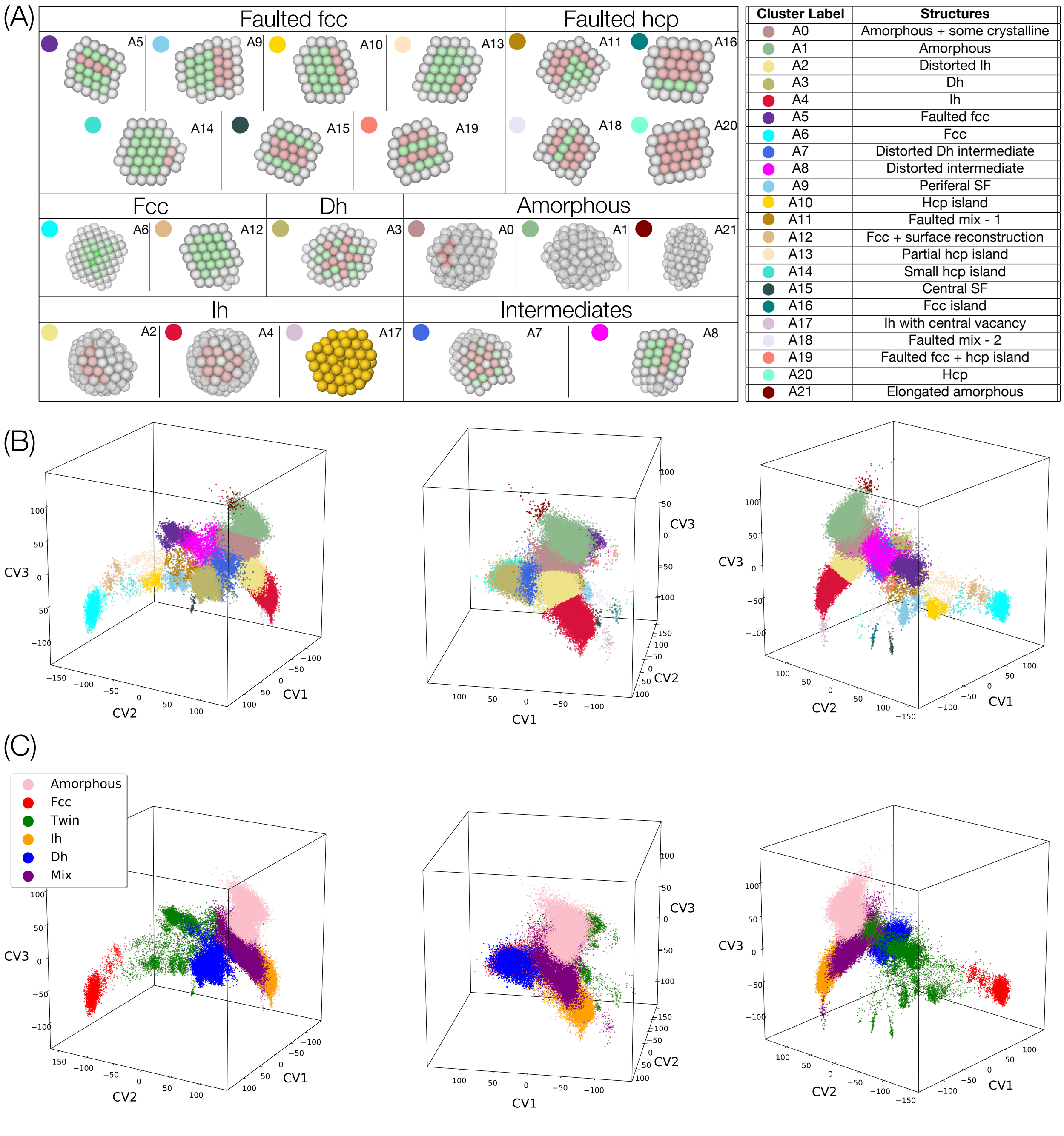

Figure S5: A) Au_{147} figures of the 22 main structural families identified via mean shift clustering. Every figure is associated to an alpha numeric label and a color. In the table on the right are reported the descriptions of each structural family. B) Different perspectives of the 3D chart of Au_{147}. Colors follow the mean shift clustering labels, as in panel A. C) Same chart of panel B, where colors now represent the CNA classification of the configurations.

Ag_{147}

Chart for Ag_{147} is reported in Fig. S6.

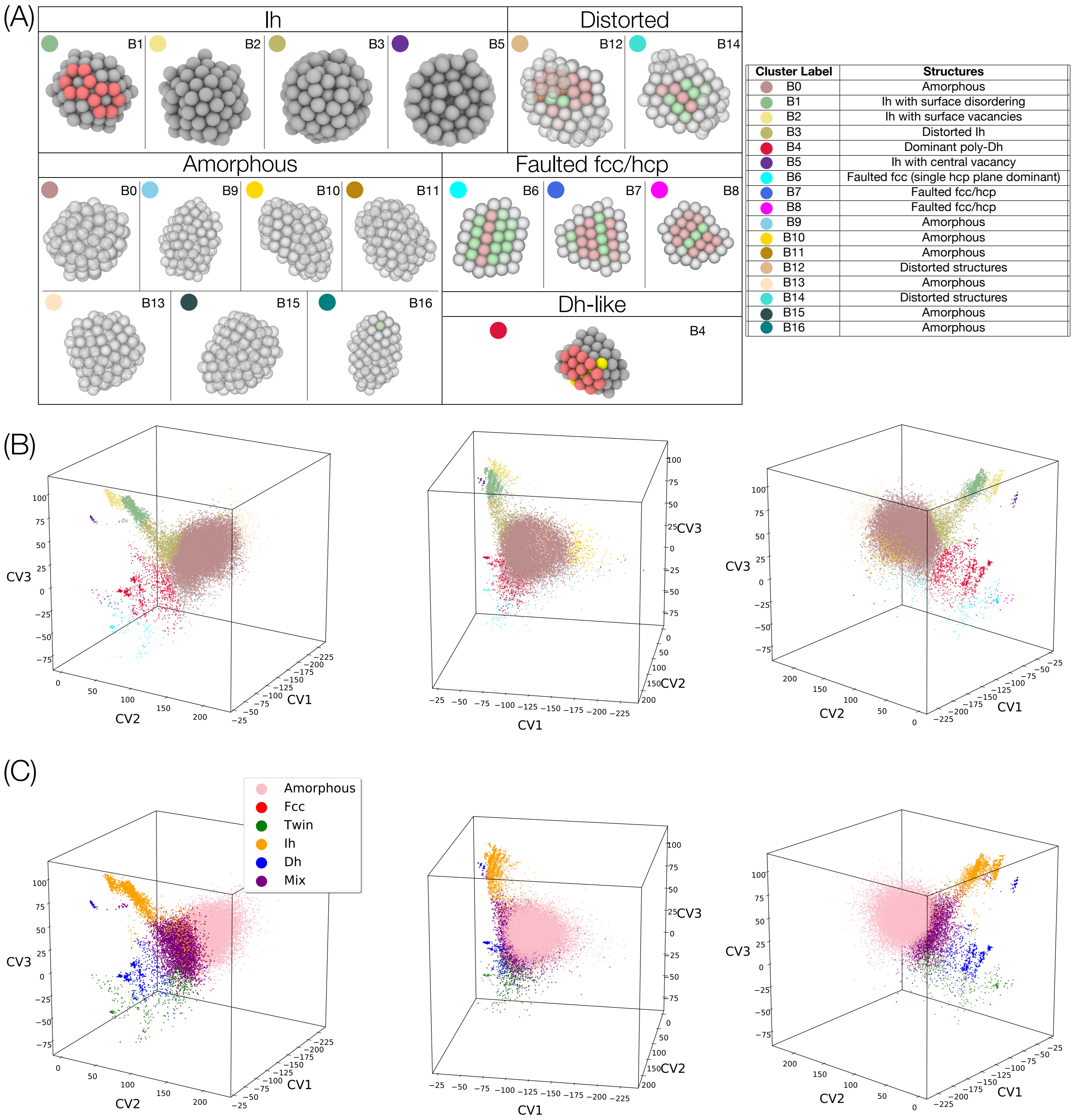

Figure S6: A) Ag_{147} figures of the 17 main structural families identified via mean shift clustering. Every figure is associated to an alpha numeric label and a color. In the table on the right are reported the descriptions of each structural family. B) Different perspectives of the 3D chart of Ag_{147}. Colors follow the mean shift clustering labels, as in panel A. C) Same chart of panel B, where colors now represent the CNA classification of the configurations.

Cu_{147}

Chart for Cu_{147} is reported in Fig. S7.

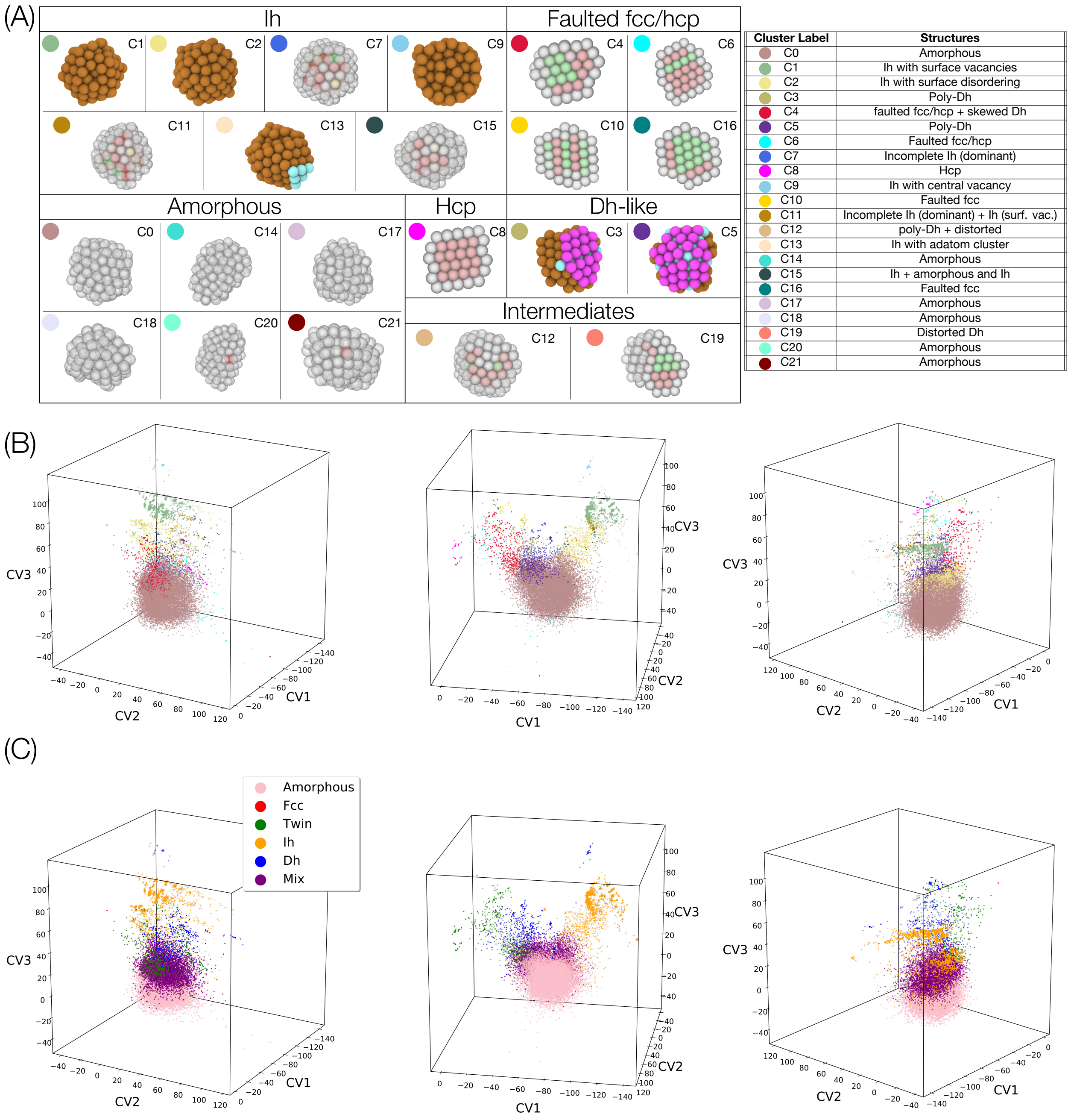

Cluster Label	Structures
C0	Amorphous
C1	Ih with surface vacancies
C2	Ih with surface disordering
C3	Poly-Dh
C4	faulted fcc/hcp + skewed Dh
C5	Poly-Dh
C6	Faulted fcc/hcp
C7	Incomplete Ih (dominant)
C8	Hcp
C9	Ih with central vacancy
C10	Faulted fcc
C11	Incomplete Ih (dominant) + Ih (surf. vac.)
C12	poly-Dh + distorted
C13	Ih with adatom cluster
C14	Amorphous
C15	Ih + amorphous and Ih
C16	Faulted fcc
C17	Amorphous
C18	Amorphous
C19	Distorted Dh
C20	Amorphous
C21	Amorphous

Figure S7: A) Cu_{147} figures of the 22 main structural families identified via mean shift clustering. Every figure is associated to an alpha numeric label and a color. In the table on the right are reported the descriptions of each structural family. B) Different perspectives of the 3D chart of Cu_{147}. Colors follow the mean shift clustering labels, as in panel A. C) Same chart of panel B, where colors now represent the CNA classification of the configurations.

Dynamical transition

CVs plots during main transitions

In Fig. S8 are reported the plots for all the three CVs in the regions highlighted in Fig. 6 of the main text.

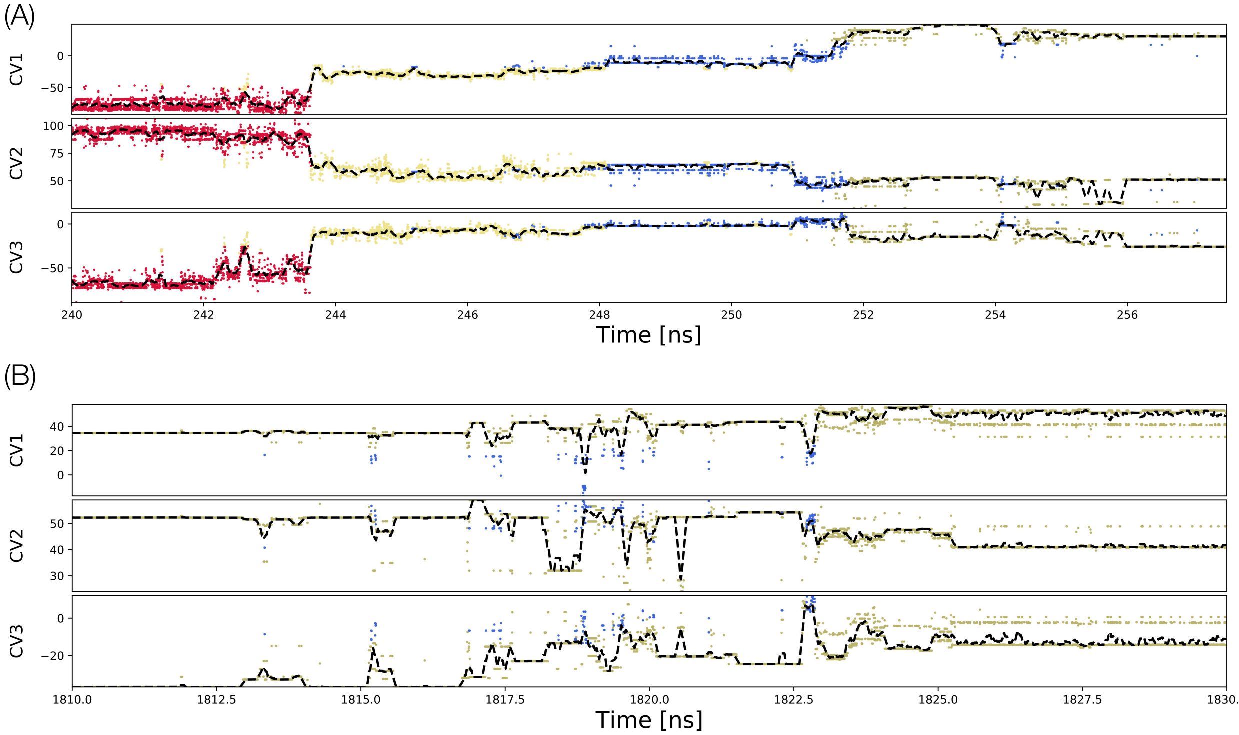

Figure S8: A) Plot of the 3 CVs versus time during the first main transition from Ih to Dh with an hcp island. B) Plot of the 3 CVs versus time during the second main transition from a Dh with hcp island to a better Dh (with no hcp island). Colors in both panels refer to the mean shift labels of Au_{147}, reported in Fig. S5.

References

(1) Pedregosa, F. et al. Scikit-learn: Machine Learning in Python. *Journal of Machine Learning Research* **2011**, *12*, 2825–2830.

(2) Kingma, D. P.; Ba, J. Adam: A Method for Stochastic Optimization. 2017.

(3) Comaniciu, D.; Meer, P. Mean shift: a robust approach toward feature space analysis. *IEEE Transactions on Pattern Analysis and Machine Intelligence* **2002**, *24*, 603–619.
